# Prevalence of Trichomonas Infection in Relation to Human Papillomavirus (HPV) in Pap Smear Samples of Female Patients Referred to Shahid Sadoughi Hospital, Yazd (Iran)

**DOI:** 10.7759/cureus.57701

**Published:** 2024-04-06

**Authors:** Malihe Azadehrah, Mahboobeh Azadehrah, Fatemeh Zeinali, Fahimeh Nokhostin

**Affiliations:** 1 Cancer Research Center, Golestan University of Medical Sciences, Gorgan, IRN; 2 Department of Obstetrics and Gynaecology, Faculty of Medicine, Shahid Sadoughi University of Medical Sciences, Yazd, IRN

**Keywords:** pap smear test, cervical tissue, uterine cervical cancer, trichomonas vaginalis, human papillomavirus

## Abstract

Objectives

Human papillomavirus (HPV) and *Trichomonas vaginalis* (TV) infections have been proposed as risk factors for cervical cancer. This study has been conducted with the aim of investigating the prevalence of TV and its relationship with HPV in women who underwent Pap smear testing as part of cancer screenings.

Materials and methods

The sampling of liquid-based cervical tissue was conducted among 500 women referred to the women's clinic of Shahid Sadoughi Hospital in Yazd, Iran. The studied samples were examined for Pap smear tests and microscopic identification of TV, as well as HPV-DNA detection and the determination of high-risk and low-risk HPV types by the polymerase chain reaction (PCR) method. The results were analyzed using the IBM SPSS Statistics for Windows, Version 24 (Released 2016; IBM Corp., Armonk, New York) software.

Results

The individuals included in the study were 16-72 years old. The prevalence rate of TV infection in this population was found to be 29.2%, and the frequency rate of HPV was reported to be approximately 19.4%, with high-risk HPV, including HPV-56, having the highest frequency. The Pap smear test results were reported as abnormal in 20.2%, and a significant correlation was observed between HPV infection and an abnormal Pap smear test (P < 0.05). In addition, a notable correlation was detected between TV infection and high-risk and low-risk HPV (P < 0.05).

Conclusion

According to the significant relationship found between the two pathogens, TV and HPV, in the abnormal Pap smear test results, TV infection can be considered a risk factor for HPV infection, as well as uterine lesions and cancer.

## Introduction

Sexually transmitted infections (STIs) are a paramount concern that significantly impacts global public health [1]. Thus, approximately 376 million infections were reported in individuals aged 15-49 in 2016 alone [2-4]. *Trichomonas vaginalis* (TV) and human papillomavirus (HPV) infections are widely recognized as the most prevalent forms of sexually transmitted diseases [5,6]. HPV is a prevalent STI that affects approximately 80% of the sexually active population globally [7,8]. Various strains of this virus have been classified. Types 16 and 18 are correlated with a heightened susceptibility to anogenital malignancies, such as penile and cervical cancers, in both males and females [3,9]. The interplay between HPV infection and other microorganisms is a critical factor in the advancement of cervical lesions toward cancer [4,10]. A potential correlation exists between HPV and other organisms, including *Neisseria gonorrhoeae*, *Chlamydia trachomatis*, and TV, which could contribute to HPV-induced harm, chronic infection, and the advancement of cancer [6,7]. TV has the potential to facilitate the progression of cervical cancer via HPV through mechanisms such as epithelial-separatory separation, immune system disruption, and inflammation [8,9,11]. These microorganisms induce chronic inflammation and have the potential to function as carcinogens through the induction of substantial cellular and molecular alterations. DNA damage and repair inhibition may result from the proliferation, recruitment, and increased production of reactive oxygen species (ROS) during the inflammatory process [12]. STIs induce alterations in the cervical epithelium that render it susceptible to mutation. These modifications accomplish this by activating oncogenes, deactivating tumor suppressor proteins, and thus enabling HPV to induce tumor lesions [13] more effectively. Inconsistent evidence exists concerning the connection between TV infection, cervical dysplasia, and cervical cancer [14]. While some publications have established a robust correlation, others do not identify TV as a potential risk factor for the development of cervical cancer [8,10,14]. The available data regarding the precise prevalence rate of TV infection in conjunction with co-infection with HPV are restricted.

Nonetheless, the development of effective strategies for the prevention and early detection of HPV and other STIs, as well as for the prevention of cervical cancer, requires epidemiological data. The inquiry regarding the frequency of TV infection in Pap smear samples obtained from female patients and its association with HPV was directed to Shahid Sadoughi Hospital in Yazd, Iran. Consequently, the objective of this research endeavor is to examine the frequency of TV infection in Pap smear samples obtained from female patients referred to Shahid Sadoughi Hospital in Yazd (Iran) in connection with HPV.

## Materials and methods

Research type and studied communities

This study is an applied study based on the descriptive cross-sectional method. The studied population included all women referred to the women's clinic of Shahid Sadoughi Hospital in Yazd, Iran, in 2022 for examination and a Pap smear test.

Sampling and sample size

In this study, the target population was selected by a simple random method from all women referred to the women's clinic of Shahid Sadoughi Hospital in Yazd in 2022 for examination and a Pap smear test. The sample size was determined by calculating P = 0.03, d = 0.05, and α = 0.05 and using the sample size formula for descriptive studies. In this study, considering that 447 people were obtained by calculating the probability of 10% attrition during the study, the final sample size was estimated to be 500 people. All the cases of invasive cervix cancer were excluded.

Data collection tool

In this study, a researcher-made form was used to collect the information of the participants. This form consists of two parts. The first part included the demographic information of the patients, and the second part included the information obtained from the results of the tests performed on the Pap smear sample. Additionally, the validity and reliability coefficient of the mentioned form were calculated based on Cronbach's alpha, which is equal to 0.9. This indicates the appropriate validity of this form.

Data collection procedure

This study is based on a descriptive cross-sectional method. After obtaining approval from the Research Center of the Faculty of Medical Sciences and Medical Health Services at Shahid Sadoughi University of Medical Sciences and the ethics committee in medical research (approval number IU/7834/2021), a simple random sampling method was performed to select 500 female patients who were referred to the women's clinic of Shahid Sadoughi Hospital in Yazd in 2022. To collect samples, the patient was positioned in the lithotomy position on the examination bed, and a sterile metal speculum was inserted. Following a visual examination of the cervix, a Pap smear sample was obtained using a cytobrush and evaluated using the Bethesda system. A liquid-based cervical tissue sample was sent to the laboratory for a Pap smear test, identification of HPV infection (high-risk and low-risk types), and TV infection. A temperature of 5-6 °C was used to treat 20 mg of the prepared tissue sample with 100 µL of proteinase K enzyme for two hours. This was done to kill any cellular accumulations that had formed. The DNA extraction was done using an AmpliSens DNA purification kit (Moscow, Russia) in accordance with the standard protocol provided with the kit. Then, papillomavirus typing was conducted using the MolecuTech REBA HPV-ID polymerase chain reaction (PCR) kit (Yongin si, South Korea), following the standard protocol included in the kit. In order to identify the protozoan TV, after sampling, the samples were transferred to the laboratory in sterile tubes containing a transport medium to prevent the protozoan from increasing the sensitivity rate of the test. Then, the samples of wet Pap smears were prepared and subjected to direct microscopic examination to identify the TV parasite.

Data analysis method

The data analysis was performed using Kolmogorov-Smirnov, Student’s *t*-test, Mann-Whitney, Pearson's correlation coefficient, and linear regression statistical tests. The confidence level in all the tests was equal to 95%, and the significance level was considered less than 0.05%. The data analysis was conducted using the IBM SPSS Statistics for Windows, Version 24 (Released 2016; IBM Corp., Armonk, New York) software.

Ethical considerations

This research adhered to the general principles of the code of ethics in research approved by the Ministry of Health and Medicine of Iran. Patients were assured that their information would be kept confidential in accordance with the Helsinki Treaty, ensuring that it would only be used for the research’s intended purposes. Additionally, the patients did not incur any additional costs as a result of the implementation of this research, and before beginning the study, the patients provided their consent. The patients were also free to discontinue the study at any time. We have used the highest-quality relevant devices in the field of sampling.

## Results

This study examined cervical cytology samples from 500 women who went to the obstetrics and gynecology clinic at Shahid Sadoughi Hospital in Yazd. The samples were used to find high-risk and low-risk types of HPV infections and TV infections. The patients studied in this research were aged 16-72 years, of which 431 people (86.2%) were under 50 years old and 69 people (13.8%) were over 50. Among these, 37.8% of them were smokers (Table 1). Among the studied patients, 101 patients (20.2%) had abnormal results in their Pap smear tests, and 399 patients (79.8%) had a routine Pap smear test.

**Table 1:** The demographic characteristics and Pap smear test results of the studied patients

Variables	Total number, N (%)
Age	
Under 50 years old	431 (86.2%)
Over 50 years	69 (13.8%)
Education	
Illiterate	25 (5%)
Elementary	194 (38.8%)
Diploma	121 (24.2%)
Academic	157 (31.4%)
PhD	3 (0.6%)
Smoking use	
Yes	189 (37.8%)
No	311 (62.2%)
Pap smear test	
Abnormal	101 (20.2%)
Normal	399 (79.8%)

After the identification of HPV infection by PCR test and subsequent determination of the specific type of infection, it was found that among the 500 samples examined, 97 (19.4%) tested positive for HPV infection. Among these, 38 samples (7.6%) were found to be infected with high-risk HPV infection, and 59 samples (11.8%) were infected with high-risk infection. The highest frequency of low-risk HPV infection was related to HPV-6, occurring in 3.2% of all the studied patients. Among high-risk HPV types, HPV-56 infection showed the highest frequency, occurring in 2% of patients.

In examining the relationship between high-risk and low-risk HPV infections with age, it was found that the frequency of individuals testing positive for HPV in the age group over 50 years was 6.3%, and it was 15.9% in the age group under 50 years. Based on the chi-square test, it was determined that there is a significant association between high-risk and low-risk HPV infection and the age variable (P < 0.05). Specifically, as age increases, the risk of high-risk and low-risk HPV infection also increases (Table 2).

**Table 2:** The frequency distribution of high-risk and low-risk HPV infection based on age group in the studied patients HPV: human papillomavirus

Variables	Total number, N (%)		Chi-square	P-value
	Positive N (%)	Negative N (%)		
High-risk HPV			7.9	0.005
Under 50 years old	27 (6.3%)	404 (93.7%)		
Over 50 years old	11 (15.9%)	58 (84.1%)		
Low-risk HPV			9.9	0.002
Under 50 years old	43 (10%)	388 (90%)		
Over 50 years old	16 (32.2%)	53 (76.8%)		

Furthermore, the chi-square statistical analysis showed a significant relationship between high-risk and low-risk types of HPV infection with Pap smear test results (P < 0.05) because HPV infection causes changes in the epithelial cells of the cervical tissue, which in turn affects its cytology (Table 3). Among the 59 patients with positive LR-HPV, 20 patients (33.9%) had abnormal Pap smear results. Similarly, among the 38 patients with positive HR-HPV, 21 patients (55.3%) had abnormal Pap smear results.

**Table 3:** The frequency distribution of high-risk and low-risk HPV infection based on Pap smear test results in the studied patients HPV: human papillomavirus; HR: high risk; LR: low risk

Variables	Abnormal Pap smear, N (%)	Normal Pap smear, N (%)	Total sum, N (%)	P-value
Positive LR-HPV	20 (33.9%)	39 (66.1%)	59 (100%	0.005
Negative LR-HPV	81 (18.4%)	360 (81.6%)	441 (100%)	
Total sum	101 (20.2%)	399 (79.8%)	500 (100%)	
Positive HR-HPV	21 (55.3%)	17 (44.7%)	38 (100%)	0.000
Negative HR-HPV	80 (17.3%)	382 (82.7%)	462 (100%)	
Total sum	101 (20.2%)	399 (79.8%)	500 (100%)	

The prevalence of TV infection was found to be 29.2% in the examined samples. It was also shown that there is a significant relationship between the age variable and TV infection, based on the chi-square test and Pap smear test (P < 0.05) (Table 4). The presence of this infection leads to cellular alterations, which can potentially cause more severe effects of HPV infection and provide the basis for progression to malignancy (Table 5).

**Table 4:** The frequency distribution of TV infection based on age group in the studied patients TV: *Trichomonas vaginalis*

Variables	Total number, N (%)		Chi-square	P-value
	Positive N (%)	Negative N (%)		
Under 50 years old	120 (27.8%)	311 (72.2%)	2.7	0.09
Over 50 years old	26 (37.7%)	43 (63.3%)		

**Table 5:** The frequency distribution of positive and negative cases of TV infection based on Pap smear tests in the studied patients TV: *Trichomonas vaginalis*

Variables	Abnormal Pap smear, N (%)	Normal Pap smear, N (%)	Total sum, N (%)	Chi-square	P-value
Positive TV infection	44 (30.1%)	102 (69.8%)	146 (100%)	22.6	<0.001
Negative TV infection	57 (16.1%)	297 (83.9%)	354 (100%)		
Total sum	101 (20.2%)	399 (79.8%)	500 (100%)		

The examination of the results showed that among 146 patients who tested positive for TV infection, 19 patients (13%) had high-risk HPV infections, and these results were reported to be statistically significant (P < 0.05) (Table 6). Also, the results showed the frequency of low-risk HPV types in Pap smear test samples with TV infection. Among 146 patients with TV infection, 32 patients (29.1%) were infected with low-risk HPV infection. These results indicated a statistically significant relationship between the two infectious agents in the studied subjects (P < 0.05). These two pathogenic agents, particularly TV, have provided the basis for the incidence of HPV infection and have mutually influenced each other.

**Table 6:** The frequency distribution of TV infection based on high-risk and low-risk HPV infection in the studied patients TV: *Trichomonas vaginalis*; HPV: human papillomavirus

Variables	Positive, N (%)	Negative, N (%)	Chi-square	P-value
High-risk HPV infection				
Positive TV infection	19 (13%)	127 (87%)	19.48	<0.001
Negative TV infection	19 (5.4%)	335 (94.6%)		
Total sum	38 (7.6%)	462 (92.4%)		
Low-risk HPV infection				
Positive TV infection	32 (21.9%)	114 (78.1)	19.48	<0.001
Negative TV infection	27 (7.6%)	327 (92.4%)		
Total sum	59 (11.8%)	441 (88.2%)		

In the cytology status of Pap smear test samples with TV infection and high-risk HPV types, it was found that among the 101 patients (20.2%) who had abnormal Pap smear test results, 11 patients (10.9%) had TV co-infection and high-risk HPV types, and 14 patients (13.9%) had low-risk HPV types and TV co-infection. In other words, among 101 patients with abnormal Pap smear test results, 25 patients (24.8%) had TV co-infection and high-risk or low-risk HPV types (Table 7). Fisher's exact test revealed a statistically significant relationship between the different conditions of high-risk and low-risk HPV infection and TV, as indicated by the Pap smear test results of the patients (P < 0.05).

**Table 7:** The different types of high-risk and low-risk TV and HPV infections based on the Pap smear test results in the studied patients HPV+, TV+: positive human papillomavirus and positive *Trichomonas vaginalis* HPV-, TV+: negative human papillomavirus and positive *Trichomonas vaginalis* HPV+, TV-: positive human papillomavirus and negative *Trichomonas vaginalis* HPV-, TV-: negative human papillomavirus and negative *Trichomonas vaginalis*

	HPV+, TV+, N (%)	HPV-, TV+, N (%)	HPV+, TV-, N (%)	HPV-, TV-, N (%)	Chi-square	P-value
High-risk HPV and TV					22.6	0.001
Normal Pap smear	8 (2%)	94 (23.6%)	9 (2.3%)	288 (72.2%)		
Abnormal Pap smear	11 (10.9%)	33 (32.7%)	10 (9.9%)	47 (46.5%)		
Low-risk HPV and TV					16.6	<0.001
Normal Pap smear	18 (4.5%)	84 (21.1%)	21 (5.3%)	327 (65.4%)		
Abnormal Pap smear	14 (13.9%)	30 (29.7%)	6 (5.9%)	51 (50.5%)		

## Discussion

The current study aimed to examine the prevalence of TV infection and its association with HPV in cervical tissue samples. A total of 500 female patients referred to the women's clinic in Yazd, Iran, were included in the study. Among these patients, 86.2% were under the age of 50. Additionally, 20.2% of individuals had abnormal Pap smear test results, while 79.8% had normal Pap smear results. In our study, the prevalence rate of TV infection was reported to be 29.2%, and a significant relationship was observed between TV infection and abnormal Pap smear test results, with approximately 30.1% of patients infected with TV having abnormalities in their Pap smear test results. The frequency of high-risk and low-risk types of HPV infection in this population was reported to be 19.4% (87 positive cases). Among these, 7.6% were identified as having HPV-HR infection and 11.8% as having HPV-LR infection, but both high-risk and low-risk types of HPV had a significant relationship with age. Also, among 101 patients with abnormal Pap smear test results, 25 patients (24.8%) had TV co-infections and high-risk or low-risk types of HPV infections. HPV and TV infections are among the most common STIs worldwide, and both of them are associated with multiple health outcomes in men and women [15, 16]. TV infection can increase the risk of cervical cancer [17, 18]. The interaction between cervical cancer and TV has not yet been fully determined. Still, it is believed that the inflammatory process caused by this protozoan makes the epithelium susceptible to carcinogenesis (16).

Studies have shown that a previous history of infection with TV leads to an increased risk of HPV infection, which is mainly due to the types of viruses with a high oncogenic risk [19, 20]. It was also shown that HPV is a risk factor for TV, indicating a possible cooperation between these microorganisms that contributes to changes in the cellular microenvironment [21]. The TV, through the release of lytic enzymes, leads to the reduction of the mucous layer of the vaginal wall and, thus, the reduction of vaginal fluids. The final result of the reduction of vaginal fluids is the formation of microlesions in the epithelium, which facilitates the attachment of HPV to the epithelium and the integration of the genomic material of the virus with the DNA of the host cell. Consequently, this can result in DNA damage to the host cell and the initiation of the carcinogenesis process [22-23]. Therefore, various evaluations have been performed worldwide to investigate the rate of TV and HPV co-infection in women. In a study conducted by Cunha et al. in 2020, among the 353 sexually active women, 204 (57.8%) had HPV-DNA infection, 140 (68.6%) had HPV/STI infection, and 64 (31.4%) had only HPV infection. TV infection showed a positive relationship with HPV infection. The highest number of lesions was reported in women with HPV/co-infection (18.8%) [23]. Their study is consistent with our study in terms of the relationship between HR-HPV infections and positive TV cases.

In addition, there is a significant relationship between HPV co-infection and abnormal Pap smear test results. Thus, the occurrence of TV and HPV co-infection in these individuals can be considered a risk factor. Of course, in our study, there were no cases of cervical cancer, and no grades were determined in terms of histology. However, abnormalities were observed on the cytology slide, indicating changes in the cells of the cervical epithelial tissue. In their study, Lv et al. reported 3.1% of TV infections among people with positive HR-HPV infections. Also, the prevalence rate of TV cases can be stated as 0.2% in people with a negative HR-HPV infection [24]. Of the individuals who tested positive for HR-HPV, 13% were found to have a TV infection, while only 7.6% of those who tested negative for HR-HPV did. There was a significant relationship with age, which is similar to the findings of Lv et al. Both of the results somehow mention that the TV and HPV co-infections have synergistic effects on each other, and the changes they create in the target cells of the cervical tissue can be the basis for other infections. In a study conducted by Zhang et al., the frequency of HPV-HR infection was 7%, and TV infection was detected in 1.7% of people, indicating a high level of lower genital tract infections (LGTIs) in Beijing.

From a clinical point of view, the screening operation is important for the presence of pathogens that cause HR-HPV co-infection. Their study reported that the frequency of positive TV co-infection with HR-HPV was found to be 18.8% (three cases), and the frequency of cervical abnormality in positive HR-HPV infection was 7.8%. Compared to our study, 7.6% of HR-HPV infections were detected, and 13% had a TV co-infection. The HPV-56 and HPV-54 infection types have the highest frequency of co-infection with TV [25]. Also, in Lazenby's study, among the people with HR-HPV infections, TV infection was identified at 10.4%, with the HPV-16 and HPV-35 types having the highest frequency. Their study implies a relationship between TV and HPV [8], and our study is consistent with finding this relationship. However, in our study, other types of high-risk HPV were identified. Among these, high-risk HPV-56 infection had the highest frequency, which could be attributed to its geographical distribution in which the detection of HPV types has been effective. In general, according to the mentioned issues, it is not possible to establish a conclusive relationship between TV and cervical cancer. Still, the results of our study and other studies showed that TV is associated with HPV infection, cervical lesions, and cervical cancer. Therefore, it may be useful to follow-up patients following a TV diagnosis. All the studies somehow show that the rate of TV and HPV co-infection in women with cervical lesions is high, and it is recommended that gynecologists take into account the presence of HPV infection and cervical lesions when diagnosing TV infection. 

Limitations of the study

There are certain limitations to our investigation. We conducted the investigation exclusively at a single hospital clinic, which may limit its representativeness for the wider community. The varied characteristics or risk factors of women who visit this clinic may influence the prevalence rates of TV and HPV compared to those who do not. The cross-sectional study approach indicates that we can only identify associations between TV, HPV, and abnormal Pap smear results at a specific moment in time. This hinders our capacity to establish causality or precisely identify the connection between TV infection and HPV. We identified the presence of TV and HPV using laboratory techniques, including microscopic examination and PCR. Although these approaches are well-established, their sensitivity and specificity may limit their capacity to identify all instances of sickness.

## Conclusions

According to the results of this study, a significant relationship has been observed between TV infection and high-risk and low-risk HPV infection, which may somehow be able to inform about the effects of TV infection and its foundation for HPV infection in the cervical tissue. Furthermore, the simultaneous presence of these two pathogens can lead to more severe consequences for the infected person.
